# Congenital Partial Absence of Pericardium in an Elderly Patient

**DOI:** 10.7759/cureus.18437

**Published:** 2021-10-02

**Authors:** Antoine El Khoury, Mohamad Zayour, Carla Hanna, Miril Janji, Samer R Nasr

**Affiliations:** 1 Cardiology, University of Balamand, Beirut, LBN; 2 Internal Medicine, University of Balamand, Beirut, LBN; 3 Cardiology, Mount Lebanon Hospital, Beirut, LBN

**Keywords:** pericardium, pericardial agenesis, congenital absence of pericardium, partial absence of pericardium, congenital pericardial defect

## Abstract

Patients with a complete absence of pericardium require no intervention as they are mostly asymptomatic. Due to the risk of herniation, patients with partial absence of pericardium tend to present with symptoms and may benefit from treatment. We report a case of an elderly patient who presented for severe colitis and was incidentally found to have a partial absence of the pericardium on the right side of the heart.

## Introduction

The pericardium is a thin layer that contains the heart and covers its outer surface. In very rare cases, the pericardium may be absent due to a congenital defect [1]. It can present with either partial or complete absence [2]. It is usually detected incidentally during imaging or by autopsy [1]. It is often associated with other congenital cardiac defects such as tetralogy of Fallot or atrial septal defects [3]. Most patients remain asymptomatic and are diagnosed incidentally, making this diagnosis challenging. Echocardiography, CT scan, and magnetic resonance imaging are used for the diagnosis [1]. Patients with a complete absence of pericardium require no treatment, while patients with a partial absence of pericardium are at risk for herniation, and treatment with pericardial reconstruction is reasonable for this group [4].

## Case presentation

**Figure 1:**
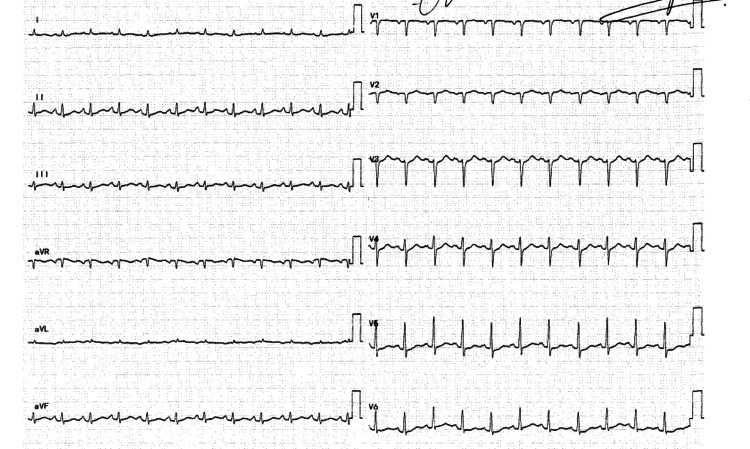
Twelve leads electrocardiogram showing sinus tachycardia, low voltage in the limb leads, and late transition of R waves in precordial leads (V4-V5).

**Figure 2:**
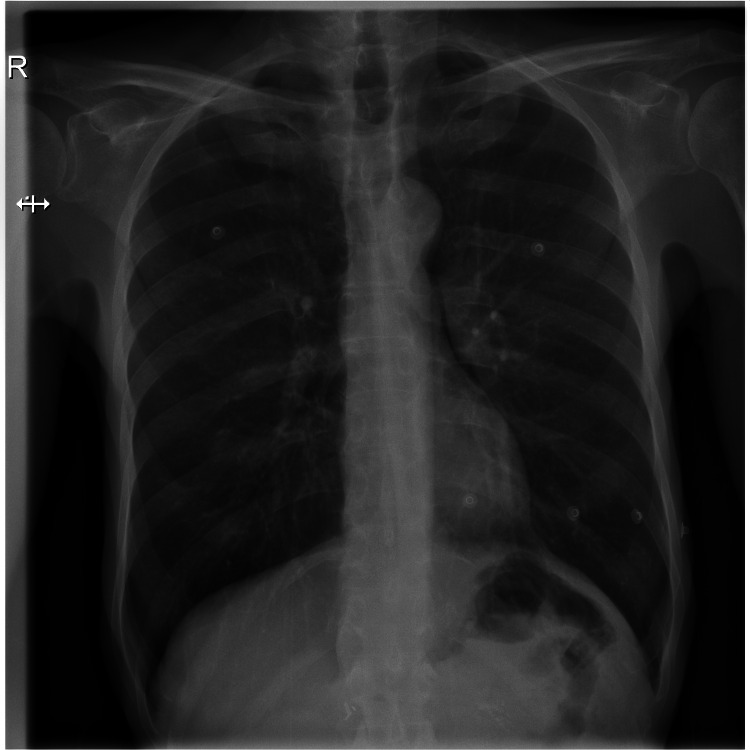
Chest X-ray showing a small heart structure.

**Figure 3:**
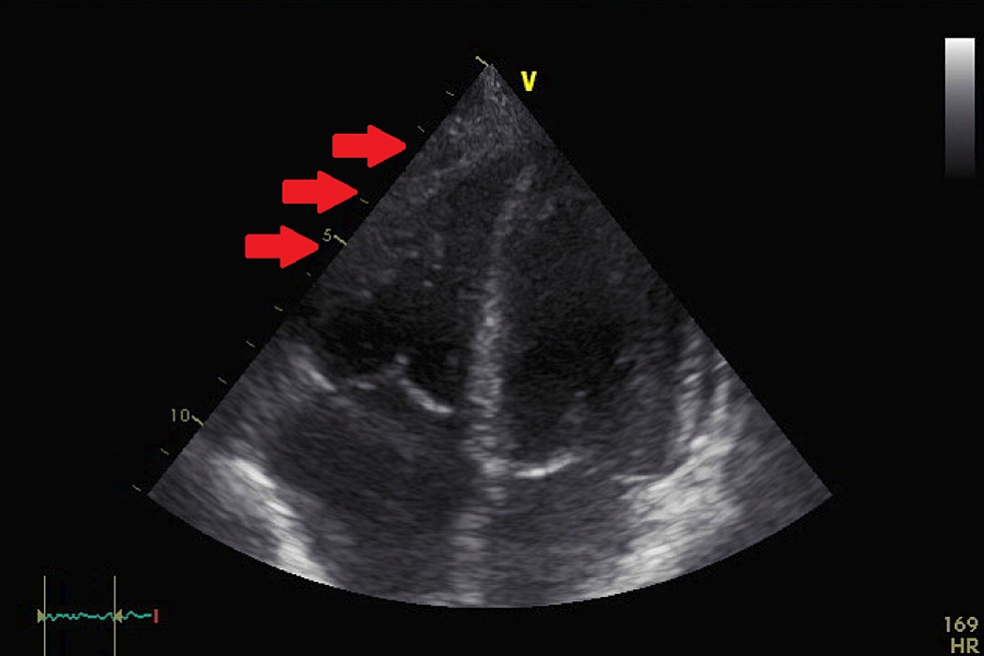
Apical four chambers view of the heart showing absence of the pericardium on the right ventricle area (red arrows).

**Figure 4:**
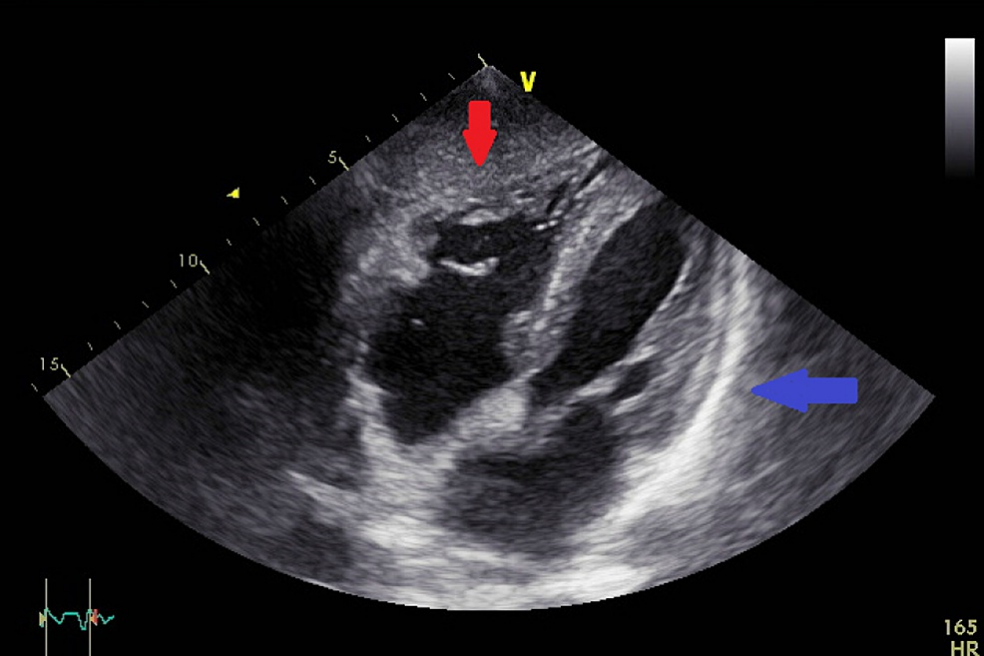
Subcostal view of the heart showing absence of the pericardium on the right side of the heart (red arrow) and presence of the pericardium over the left side of the heart (blue arrow).

**Figure 5:**
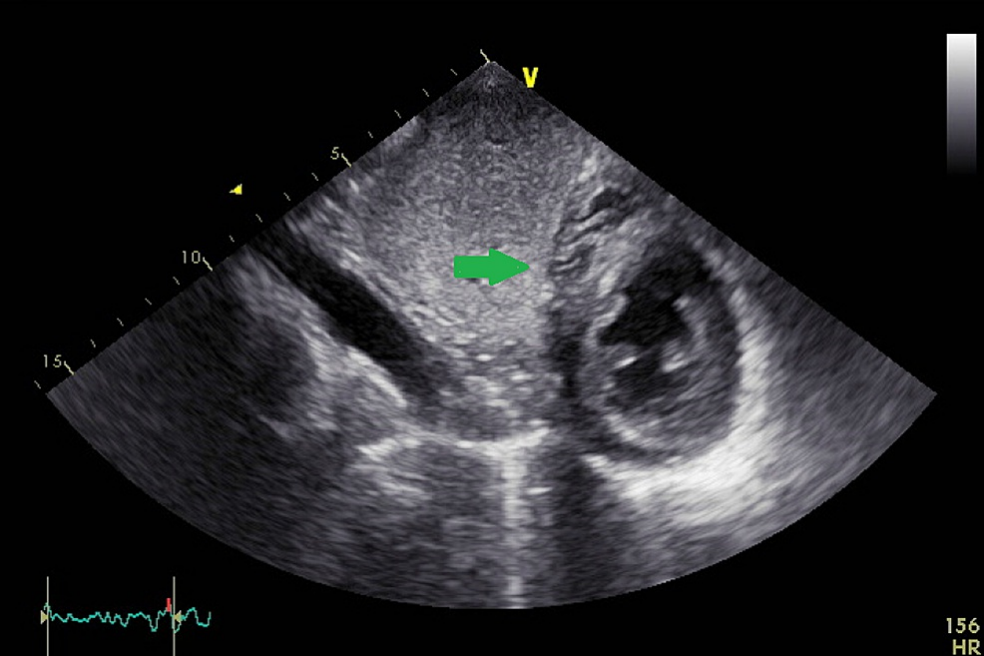
Short axis view of the heart showing absence of the pericardium over the right side of the heart (green arrow).

A 70-year-old male long-time diabetic patient presented for severe colitis, hyperglycemia, and chronic pancreatitis. His high glucose level was well treated with insulin. During this admission, the patient complained of mild chest pain and dyspnea. His initial ECG showed sinus tachycardia, low voltage in the limb leads, and late transition of R waves in precordial leads (V4-V5) (Figure 1). A chest X-ray revealed a small heart with a leftward displacement of the cardiac structures (Figure 2). A transthoracic echocardiogram was performed to assess for volume status and dyspnea, and it showed a normal systolic and diastolic function without valvular stenosis or regurgitation but detected a partial absence of the pericardium on the right side as can be seen on the subcostal view and the apical four chambers view (Figures 3, 4). A short-axis view of the heart showed similar findings (Figure 5).

**Figure 6:**
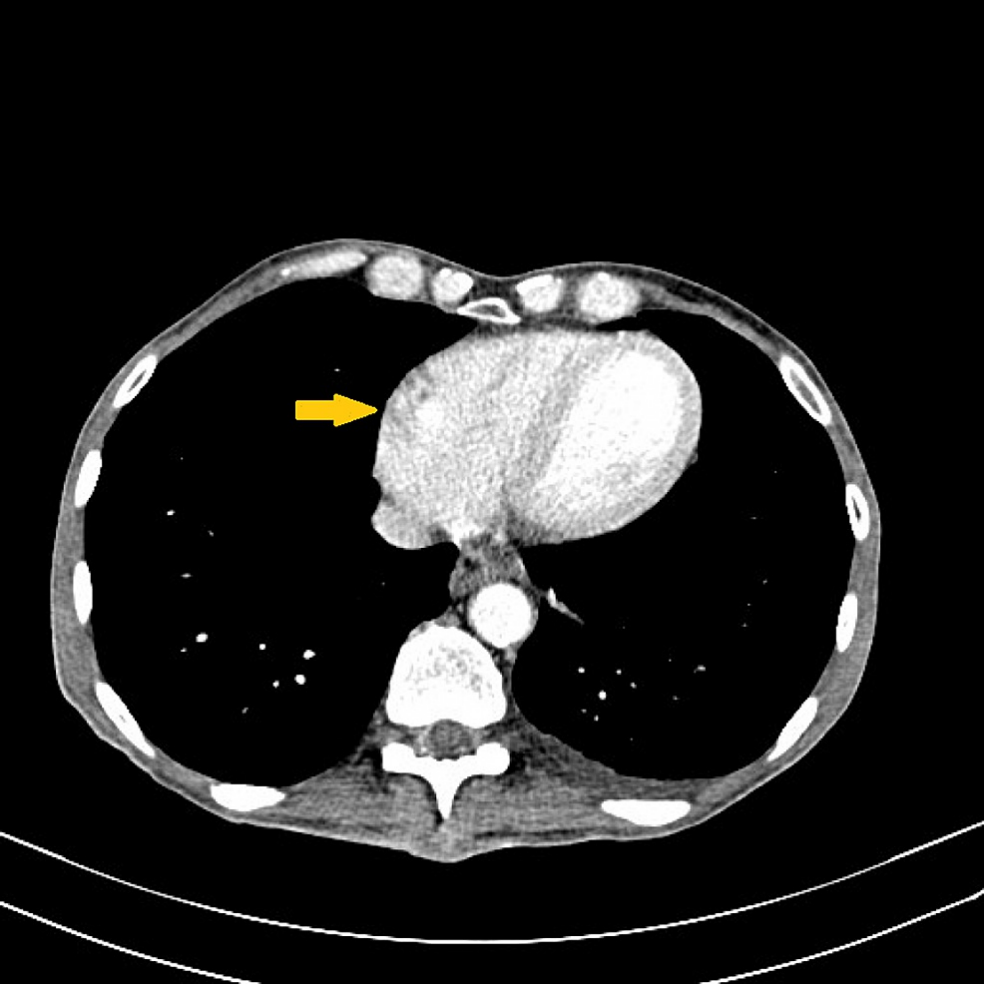
CT scan image showing the partial absence of the pericardium covering the right side of the heart (yellow arrow).

**Figure 7:**
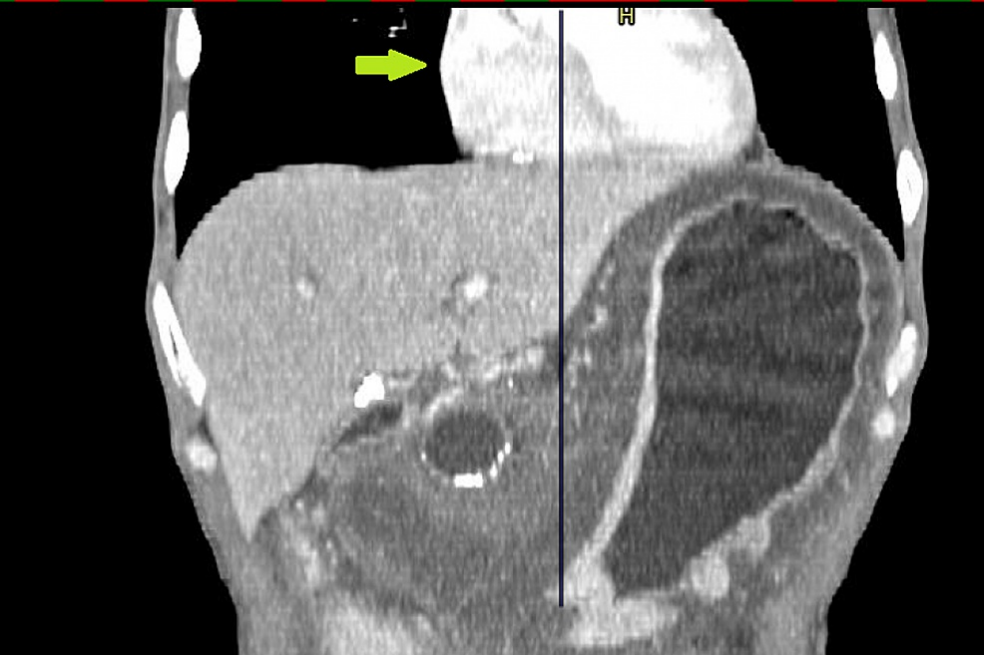
CT of coronal planes showing similar findings of absence of pericardium over the right heart (green arrow).

A CT scan of the abdomen performed on admission for investigation of chronic pancreatitis was able to detect few cuts from the chest area allowing for the evaluation of a part of the heart. It confirmed the echocardiographic findings of the pericardial absence of the right side of the heart by showing clear heart borders, unlike the aspect seen covering the left heart (Figures 6, 7). The scan showed diffuse gastric bowel wall thickening compatible with gastro-entero-colitis. 

## Discussion

During inspiration, the intra-thoracic pressure drops leading to an increase in venous return toward the right heart. This process leads to an increase in right ventricle size [5]. The pericardium expands and does not affect the left ventricle filling. It provides stability to all heart structures. In very rare cases, the pericardium may be absent due to a congenital defect [6]. It can present with either partial or complete absence [2]. It is usually detected incidentally during imaging or by autopsy [1]. It is often associated with other congenital cardiac defects such as the tetralogy of Fallot or atrial septal defects [4]. Most patients remain asymptomatic and are diagnosed incidentally, and few patients present with palpitations and chest pain [7]. The latter is due to a herniation of a heart cavity in the chest wall [8]. The heart motion increases, which predisposes patients to type A aortic dissection, but this is exceedingly rare [9]. The diagnosis can be suspected on echocardiography with images on the parasternal long-axis view may show the right ventricle to be more prominent, the heart may be shifted toward the left, which will result in a paradoxical septum [10]. These findings indicate the complete absence of the pericardium and are associated with right ventricular overload [11]. Wall motion abnormalities indicate herniation of the cavity or may indicate compression of a coronary artery [12]. Few patients develop findings resembling acute coronary syndrome and may lead to death [12]. These findings of herniation indicate a partial absence of the pericardium [8]. The diagnosis can be made based on transthoracic echocardiography alone but MRI and CT can be an added value in more obscure cases [1].

Patients with a complete absence of pericardium require no intervention as they are mostly asymptomatic, and the risk of cavity herniation and coronary artery damage is low. Due to the risk of herniation and other fatal complications, patients with partial absence of pericardium tend to present with symptoms and may benefit from treatment [4]. If the herniation is causing major defects, the pericardium can be removed in order to transform the partial defect into a complete defect resulting in a reduction of symptoms [4]. If the defect is small it can be closed in order to preserve the pericardium. Other indications for surgery include persisting symptoms that cannot be explained by other diseases. Surgery may be considered as prophylaxis to prevent herniation that may affect the coronary arteries [4]. Surgery options include pericardiectomy, patch closure of the pericardial foramen, and pericardioplasty [12-13]. A patch is a pliable, nonbiological, and immobile structure that can resist high tension from surrounding structures without being affected or damaged [4]. The procedure is accomplished by a left thoracotomy, and several patches are sutured to the posterior mediastinum and then are transmitted and fixed anteriorly [4]. This technique is aimed at stabilizing the heart to prevent herniation of heart structures and is used to treat patients with partial absence of pericardium [4]. A pericardiectomy can be performed under general anesthesia with a sternotomy. During this procedure, a part or the entire pericardium will be removed to correct the defect. The procedure consists of removing the anterior pericardium from phrenic nerve to phrenic nerve and the removal of the diaphragmatic pericardium posterior to both phrenic nerves [14]. 

The patient, in this case, did not have symptoms from his condition that is classified as congenital and present since birth. As a result, no intervention was to be performed for the moment, and the patient was scheduled for routine follow-up.

## Conclusions

The absence of pericardium is a very rare congenital disease and is diagnosed incidentally when the imaging is performed for other reasons. The pericardium can be completely absent, and this is a less risky condition. A partial absence of pericardium is more dangerous and can lead to herniation of the heart cavities, aortic dissection, and damage to the coronary arteries. If detected at a young age and considered a cause of the patient's symptoms, it should be surgically repaired. When detected later in life in the elderly population, especially if asymptomatic, interventions should be delayed because it is unlikely to cause damage at this point.
